# Conversion of a classical microbiology laboratory to a total automation laboratory enhanced by the application of lean principles

**DOI:** 10.1128/spectrum.02153-23

**Published:** 2024-01-17

**Authors:** Graça Trigueiro, Carlos Oliveira, Alexandra Rodrigues, Sofia Seabra, Rui Pinto, Yohann Bala, Monica Gutiérrez Granado, Sandra Vallejo, Victoria Gonzalez, Carlos Cardoso

**Affiliations:** 1 Department of Microbiology, Dr. Joaquim Chaves Clinical Analysis Laboratory, Lisbon, Portugal; 2 Global Medical Affairs, bioMérieux, Marcy L’Etoile, France; 3 Lab Consultancy, bioMérieux, bioMérieux SA, Madrid, Spain; 4 Lab Consultancy, bioMérieux, bioMérieux SA, Lisbon, Portugal; University Paris-Saclay, AP-HP Hôpital Antoine Béclère, Service de Microbiologie, Institute for Integrative Biology of the Cell (I2BC), CEA, CNRS, Clamart, France

**Keywords:** turnaround time, productivity, lean principles, laboratory automation, change management

## Abstract

Laboratory automation in microbiology improves productivity and reduces sample turnaround times (TATs). However, its full potential can be unlocked through the optimization of workflows by adopting lean principles. This study aimed to explore the relative impact of laboratory automation and continuous improvement events (CIEs) on productivity and TATs. Laboratory automation took place in November 2020 and consisted of the introduction of WASPLab and VITEK MS systems. CIEs were run in May and September 2021. Before the conversion, the laboratory processed about ~492 samples on weekdays and had 10 full-time equivalent (FTE) staff for a productivity of 49 samples/FTE/day. In March 2021, after laboratory automation, the caseload went up to ~621 while the FTEs decreased to 8.5, accounting for productivity improvement to 73 samples/FTE/day. The hypothetical productivity went up to 110 samples/FTE/day following CIEs, meaning that the laboratory could at that point deal with a caseload increase to ~935 with unchanged FTEs. Laboratory conversion also led to an improvement in TATs for all sample types. For vaginal swabs and urine samples, median TATs decreased from 70.3 h [interquartile range (IQR): 63.5–93.1] and 73.7 h (IQR: 35.6–50.7) to 48.2 h (IQR: 44.8–67.7) and 40.0 h (IQR: 35.6–50.7), respectively. Automation alone was responsible for 37.2% and 75.8% of TAT reduction, respectively, while the remaining reduction of 62.8% and 24.2%, respectively, was achieved due to CIEs. The laboratory reached productivity and TAT goals predefined by the management after CIEs. In conclusion, automation substantially improved productivity and TATs, while the subsequent implementation of lean management further unlocked the potential of laboratory automation.

**IMPORTANCE:**

In this study, we combined total laboratory automation with lean management to show that appropriate laboratory work organization enhanced the benefit of the automation and substantially contributed to productivity improvements. Globally, the rapid availability of accurate results in the setting of a clinical microbiology laboratory is part of patient-centered approaches to treat infections and helps the implementation of antibiotic stewardship programs backed by the World Health Organization. Locally, from the point of view of laboratory management, it is important to find ways of maximizing the benefits of the use of technology, as total laboratory automation is an expensive investment.

## INTRODUCTION

Compared to general hematology and biochemistry laboratories, automation was late to arrive in microbiology laboratories because of the complexity of the processes involved (1–3). The most important driver of automation adoption in microbiology laboratories in recent times is represented by a shift of paradigm in clinical microbiology that increased the demand for rapid and accurate results (1, 2, 4). The availability of such results is important for the success of patient-centered approaches to treat infections and for antibiotic stewardship programs (5). Over the past 20 years, and especially since the onset of the coronavirus disease 2019 (COVID-19) pandemic, microbiology laboratories have been increasingly adopting technologies and processes that improve quality and efficiency, alongside a reduction in sample turnaround times (TATs) (1).

In total laboratory automation systems, all main system components, i.e., plating/streaking unit, incubation unit, high-resolution imaging, colony picking for identification and antimicrobial susceptibility testing, and post-imaging analysis workstation are robot driven. Artificial intelligence has recently been incorporated into imaging analysis to automatically interpret plates. Automating analyses has been the key to improving productivity (6–8). System components may be connected via a conveyor belt system or placed in convenient locations in laboratories (9). In the WASPLab system, plates are sorted out to stackers and transferred to workstations by technologists. The systems must be flexible to accommodate several variables, such as specimen types and non-standardized containers. Moreover, some samples are sent to be screened for a specific pathogen (e.g., throat swab samples to be screened for *Streptococcus pyogenes*), while others may require a variety of microbial culture and identification methods for a full diagnostic work-up (1).

There are numerous advantages to total laboratory automation. Although a high investment is needed for the conversion from a manual to an automated lab, in the long run, automated laboratories are cheaper to run (4). The implementation of total laboratory automation increases efficiency, measured as turnaround time and total productivity, and improves the quality of testing, thanks to the standardization and optimization of the procedures. Automation offers a possibility of better sample management and traceability, often requires lower volumes of samples, renders laboratory accreditation easier to obtain, and lowers the biological risk to the operators (2, 10). In the context of a microbiology laboratory, a significantly better recovery of pathogens, defined as a greater number of unique colony morphologies, a higher proportion of discrete colonies, and the identification of more species/plates, can be achieved [reviewed in (1)].

However, there are two principal elements of laboratory automation: hardware and workflow (11). Total laboratory automation improves efficiency through the reduction of repetitive tasks with moderate added value, i.e., through the modification of the workflow. Automated incubators with digital imaging drastically reduce the number of manipulations of the culture media plates. About 97% of samples are suitable for automatic processing (2). Employees often resist change. Fear of the unknown, lack of trust toward leaders, comfort derived from a familiar routine, lack of perception that a change is needed, perceived lack of knowledge or competence and the necessity to retrain, poor communication of what will happen from the management, and exhaustion or saturation when changes are too frequent are all factors at the basis of resistance to change (12). Therefore, to be successful, the introduction of a change must be “managed” through a process known as change management. Change management occurs through steps (prepare, implement, monitor, sustain, and reevaluate) (12). In particular, a continuous improvement in streamlining workflow must be introduced. Change management is done with the use of lean methodology, an evidence-based approach to increase quality and efficiency that combines philosophy, processes, people, and structures (13).

The study aimed to assess the relative impact of laboratory automation, change management, and continuous improvement events (CIEs) on key performance indicators [KPIs, i.e., full-time equivalent (FTE)/day and TAT] in a clinical microbiology laboratory concerning the processing of urine samples, vaginal swabs, and stool samples that comprise over 90% of the caseload of the laboratory.

## RESULTS

### Pre-conversion

#### Caseload and FTE

In May 2019, prior to the conversion, Dr. Joaquim Chaves Saúde Clinical Analysis Laboratory (JCS) processed on average 492 samples/day on weekdays, 389 on Saturdays, and ~12 on Sundays. Three-quarters of the samples received were urine samples, 16% were vaginal swabs, 4% stool samples, 2% pharyngeal swabs, and ~1% were samples sent in for mycology testing. Other sample types comprised 3%. The laboratory had 10 FTEs of staff at the time.

#### KPIs

The productivity pre-conversion was 49 samples/FTE/day (Table 1). The median TAT was the shortest for the negative urine samples [median 13 h; interquartile range (IQR; 3.1; 23.7)] and the longest for the positive stool samples [median 93.3 h; IQR (70.1; 122.8)]. Positive urine and vaginal swabs, in comparison, had a similar median TAT with a narrower IQR for vaginal swabs [median 73.7 h; IQR (43.4; 94.6) versus median 70.3 h; IQR (64.5; 93.1)]. Full results are given in Table 2.

**TABLE 1:** Productivity key performance indicator at the three timepoints of the study and improvements achieved

Key performance indicator	Pre-conversion (PRE)	Post-automation (POST)	% Change from PRE	Post-automation and CIEs	% Change from POST	Predefined goal	Overall improvement (%)
# of average daily samples; FTE	492; 10	621; 8.5		935^*a*^; 8.5		1,000; 10 or 850; 8.5	NA
Productivity (samples/FTE/day)	49	73	49	110	51	100	124

^*a*^ Hypothetical caseload that current FTE would be able to process; CIE, continuous improvement event; FTE, full-time equivalent; IQR, interquartile range; Q1, first quartile; Q3, third quartile.

**TABLE 2:** Turnaround time key performance indicator at the three timepoints of the study and improvements achieved^*c*^

	Pre-conversion (PRE)			Post-automation (POST)			% Change from PRE	Post-automation and CIEs			Predefined goal	% Change from POST	Overall improvement (%)	TAT IQR reduction (%)
Urine samples (*N*; positivity rate)	10,404; 21%			11,230; 20%				9,981; 24%						
	Median	Q1	Q3	Median	Q1	Q3		Median	Q1	Q3				
Negative	13.2	3.1	23.7	21.3^*b*^	21.2	22.9	−61	19.1^*b*^	18.6	20.8	20	10	−47	89
Positive	73.7	49.4	74.6	47.4^*b*^	43.5	68.7	36	40.0^*b*^	35.6	50.7	40	16	46	67

Vaginal samples (*N*; positivity rate)	3,951; 13%			3,381; 12%				2,959; 13%						
	Median	Q1	Q3	Median	Q1	Q3		Median	Q1	Q3				
Negative	48.5	45.0	68.6	45.7^*b*^	43.7	62.6	6	44.4^*b*^	41.8	48.4	45	3	8	72
Positive	70.3	63.5	93.1	62.2^*b*^	46.1	85.1	12	48.2^*b*^	44.8	67.7	60	23	31	23

Stool samples (*N*; positivity rate)	1,126; 3%			1,346; 2%				1,573; 3%						
	Median	Q1	Q3	Median	Q1	Q3		Median	Q1	Q3				
Negative	68.1	48.2	74.5	52.9^*a*^	47.4	76.4	22	48.5^*b*^	43.8	69.3	50	8	29	3
Positive	93.3	70.1	122.8	69.5	67.3	94.4	26	70.1	49.9	92.5	65	−1	26	19

^*a*^ *P* < 0.05.

^*b*^ *P* < 0.0001 for median comparison between two consecutive observation periods.

^*c*^ CIE, continuous improvement event; IQR, interquartile range; Q1, first quartile; Q3, third quartile.

### Post-automation

#### Caseload and FTE

In March 2021, following the introduction of automation, the average number of samples in a day processed by JCS equaled 621 on weekdays, 417 on Saturdays, and ~11 on Sundays. The distribution of sample types changed only slightly. Seventy-eight percent of samples received were urine samples, 12% were vaginal swabs, 2% were stools, and 1% were pharyngeal swabs, and ~1% were samples sent in for mycology testing. Other sample types comprised 6%. In March 2021, the availability of FTEs in the laboratory went down to 8.5 (Table 1).

#### KPIs

Given the average number of samples received in a day and the reduced FTEs, the productivity of the JCS laboratory increased to 73 samples/FTE/day (Table 1). The automation led to a decrease in TAT for all sample types except for negative urine samples due to the discontinuation of the UF1000i urinalysis system, whereas the decrease by 26% of the TAT for positive stool samples did not reach statistical significance (*P* = 0.121). Nonetheless, the median TAT was still the shortest for the negative urine samples [median 21.3 h; IQR (21.2; 22.9)] and the longest for the positive stool samples [median 69.5 h; IQR (67.3; 94.4)]. It is important to mention that for the urine workflow, the removal of the screening step using the UF1000 explains the longer TAT for negative urine, since currently, the sample status depends on the interpretation of culture on agar. TAT for positive urine samples went down to a median of 47.4 h (43.5; 68.7), while positive vaginal swabs decreased to a median of 62.2 h (46.1; 85.1) (Table 2).

### Post-automation and CIEs

#### Caseload and FTE

In September 2021, the average number of samples processed and the available FTEs remained unchanged since March 2021.

#### KPIs

Automation, together with continuous improvement events, increased productivity to a theoretical level of 110/FTE/day, equivalent to processing 935 samples/day with the same FTEs, which more than doubled the original productivity observed pre-conversion (Table 1). Further improvement of median TAT was observed for positive urine [median 40.0; IQR (35.6; 50.7)] and vaginal swab (median 48.2; IQR [44.8; 67.7]) samples, while no additional improvement was seen for the positive stool samples. Predefined goals were reached for all samples except for positive stool specimens (Table 2).

The introduction of WASPLab and VITEK MS together with the CIEs led to a reduction in variability of TAT shown as a decrease in the IQR range (Table 2). For the negative samples, an IQR reduction of 89%, 72%, and 3% was observed for urine, vaginal swab, and stool specimens, respectively, while the values equaled 67%, 23%, and 19% for the positive counterparts.

### The relative impact of automation and CIEs on overall reduction in TAT

Laboratory conversion (automation and CIEs) led to a 124% improvement in productivity and improved TATs by a value ranging from 8% (for negative vaginal swabs) to 46% (for positive urine samples). TAT for positive vaginal swabs and stool samples improved by 31% and 26%, respectively (Table 2).

## DISCUSSION

**Fig 1:**
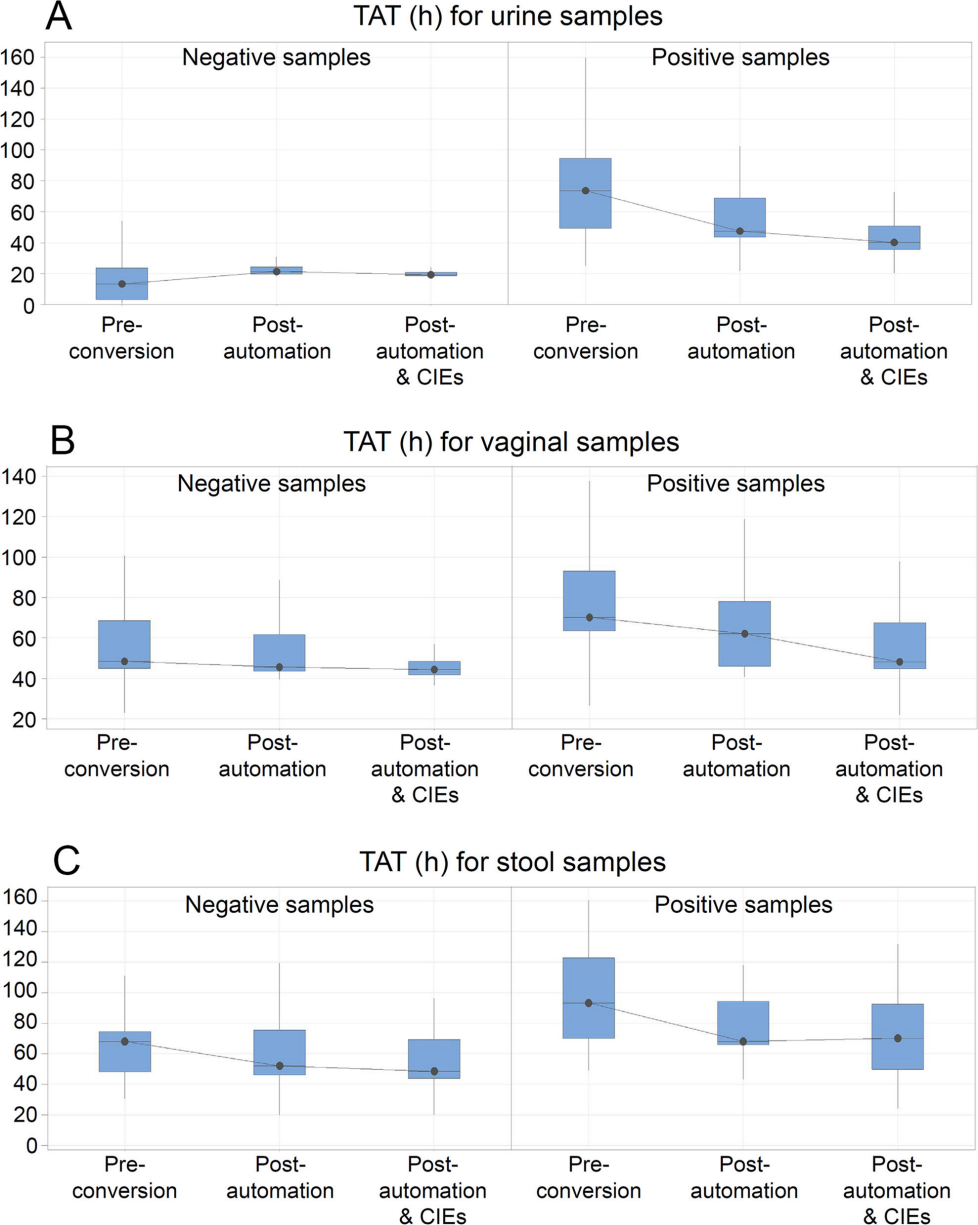
Improvement of TAT according to sample type. (A) Urine samples; (B) vaginal swabs; (C) stool samples.

The conversion of the JCS laboratory, consisting of laboratory automation facilitated by change management, substantially improved the productivity and TATs of the laboratory and reached goals predefined by the management. The improvement was the greatest for the positive urine samples, for which the median TAT decreased from just over 3 days (73.7 h) to well under 2 days (40 h, Fig. 1). The discontinuation of the UF1000 automated urine particle analyzer caused the expected lengthening of TAT for negative urine samples, although the post-conversion median TAT met the expected goal predefined by the JCS management. Moreover, TAT variability decreased following laboratory automation and CIEs. This occurred through an improvement of the standardization of the processes and full alignment with the pre-established priorities.

A more streamlined automatic workflow that eliminated many manual tasks provided the major contribution to TAT reduction; however, CIEs maximized the potential benefit of laboratory automation and contributed substantially to productivity improvements. In 2019, the lab was processing, on average, 492 samples a day with 10 FTEs; after laboratory automation, the lab was processing 621 samples a day with 8.5 FTEs. After CIEs, with 8.5 FTEs, the lab could process 935 samples per day. In 2019, 19 FTEs would have been needed to process a daily caseload of 935 specimens, whereas after the integration of laboratory automation, 13 FTEs would have been needed. In other words, had there been a caseload of 935 at the time, the improvement in productivity observed following lab conversion and optimization of workflows would have allowed for the saving of six FTEs attributable to laboratory automation alone, and of an additional 4.5 FTEs made possible by the application of lean principles (i.e., the CIEs).

The delivery of the change management program in JCS helped the laboratory staff to increase their level of understanding of automation. The change management program focused on defusing reluctance to technological changes, motivated the adoption of WASP and WASPLab technologies, developed confidence toward hardware and software systems, envisioned daily work with automation, worked on the understanding that skills are transformed and not lost, and clarified the expectations and decisions to be made within the laboratory. The procedure optimization phase based on lean management application allowed the team of the Department of Microbiology at the JCS to develop a mindset of continuous improvement that is needed to maximize the potential of laboratory automation, reach preset goals, and to sustain them, which is a fundamental element of lean management (14).

The results of this study confirm the findings published by others. In the setting of a microbiology laboratory, laboratory automation was shown to improve efficiency (15–22). In addition, the combination of matrix-assisted laser desorption ionization-time of flight mass spectrometry (MALDI-TOF)-based microbial identification and total laboratory automation individually and together improved TATs in microbiology (23, 24). Da Rin and colleagues showed that the integration of the WASPLab diagnostic microbiology system into a pre-existing system of total automation, together with resource standardization and optimization, shortened TAT (25). In a Chinese study, total laboratory automation was accompanied by the setting up of three working shifts, as opposed to a single working shift pre-automation. Together, the two actions led to a statistically significant shortening of TATs, especially for the cerebrospinal fluid sample diagnostics (26).

Unlike Cherkaoui and colleagues, who saw only a trend toward shorter median TATs, our intervention led to a substantial improvement in TAT for positive urine samples. This result strongly supports the notion that automation must be matched by laboratory work organization, which was a limitation acknowledged by Cherkaoui et al. in their study (27). In our study, laboratory automation alone was responsible for 37.2% and 75.8% reductions in TAT observed for positive vaginal swabs and positive urine samples, respectively, while the remaining 62.8% and 24.2% reduction in TAT for these samples was achieved due to CIEs. The study by Yarborough et al. confirmed that changes in urine sample workflow were necessary to maximize the efficiency of laboratory automation and to optimize TAT (28).

Taken together, the conversion of the JCS laboratory resulted in several benefits. The first benefit was the occurrence of fewer errors and associated rework. Most of the laboratory errors occur during the extra (pre- and post-) analytical phases of the testing process (29). Indeed, in the workflow before the conversion, there was a lot of room for errors since some of the samples were labeled manually and test results were manually fed into the laboratory information system. Secondly, the conversion led to fewer uncertainties concerning operating procedures, which in turn counteracted the feeling of being overwhelmed, a sensation that may be experienced by laboratory staff after a major change to the working routine. Thirdly, during the conversion, an environment favorable to continuous improvement was created, with clear procedures allowing any problem or deviation to be easily spotted. In addition, sample type-based CIEs enabled focused collaboration of the technologists and sequential validation. Such sample type-based optimization was also done by Cherkaoui et al. in Geneva (30). Lastly, productivity improvements facilitated qualified staff transfer to clinical activities despite working in the context of the COVID-19 pandemic, which led to a 20% caseload increase.

At the level of global trends, the introduction of laboratory automation and CIE helps counteract problems with staffing experienced by most of the clinical laboratories worldwide due to accelerated rates of microbiologists’ retirement and fewer people entering the field of laboratory medicine. A very recent study showed that up to 80% of microbiology laboratories have vacancies and struggle to fill them (31). There is also the phenomenon of declining reimbursement, and total laboratory automation is a way of obtaining cost savings (32).

There is one main limitation to the study, which is that it is the experience of one center. While laboratory automation has been demonstrated to provide benefits regardless of site size/organization (4), the magnitude of the benefit and impact of CIEs are likely to be site-dependent since they require finding the right combination of people, processes (workflow), and technology to unlock new sources of improvement (11). Moreover, the productivity index used in the study is strongly linked to the relative local distribution of sample types, which at the JCS laboratory was 74% urine samples, 16% vaginal swabs, 4% stool samples, and 6% other specimens. Consequently, the improvements in productivity observed after lab automation and after CIEs would be expected to vary with a different sample distribution.

A minor limitation of the study is the fact that the workflow for positive stool samples did not reach the predefined TAT goal of 65 h. However, stool samples represent only 4% of the caseload, and only 5% of them are positive, which makes the standardization of the workflow for these isolated specimens difficult. Furthermore, while previous studies by Cherkaoui and colleagues also highlighted the potential of automation to reduce TAT for other critical samples such as blood culture (33), the caseload for this sample type in the JCS laboratory was too low to be included in the present study. Moreover, the present study looked at the impact of a bundled intervention, i.e., implementation of WASPLab and VITEK MS systems. While the decrease of TAT is most likely caused by the combination of both technologies, the relative impact of each of the changes was not assessed.

In conclusion, JCS conversion resulted in substantial improvements in KPIs. While automation alone substantially improved TAT and productivity, the subsequent implementation of lean management further unlocked the potential of laboratory automation through the streamlining of the processes involved. Together, they led to the achievement of predefined goals. Future studies will be needed to see how additional developments in the field of automation, e.g., automatic colony picking for identification by MALDI-TOF (34) and antimicrobial susceptibility as well as novel algorithms for automated reading and interpretation of plates (6–8), could further improve productivity and the quality of results.

## MATERIALS AND METHODS

### The project

The project took place at the JCS in Lisbon, Portugal between May 2019 and October 2021. In 2019, a full assessment of the laboratory workflow and performance was made to size the instruments’ needs and to propose a lean design for the future microbiology laboratory, considering the different types of workflows of the laboratory. A decision was made to automate the laboratory and remove the UF1000 automated system for urine particle analysis (Sysmex, Japan). The latter move was dictated by the desire to simplify and streamline the processing of this specimen type.

In November 2020, the laboratory moved to the new facilities, where WASPLab (Copan Diagnostics Inc.) for automated specimen processing and reading and VITEK MS (bioMérieux, France), a MALDI-TOF-based microbial identification system, were installed. For urine samples, a steady routine state was achieved by March 2021. For stool and vaginal swab samples, such a routine was reached by May 2021.

**Fig 2:**
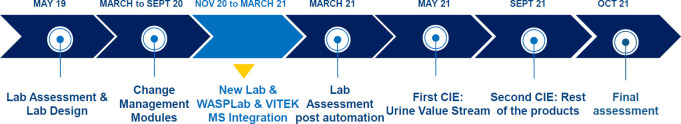
Chronology of conversion and improvement through continuous improvement events.

In May 2021, the first CIE event took place and concentrated on the urine workflow. Four months later, in September 2021, a second CIE was conducted for stool and vaginal swab samples. The final assessment of the KPIs was performed in October 2021. The chronology of the project is shown in Fig. 2. Across the three timepoints, the laboratory processed samples during similar opening times from 8 am to 10 pm in two shifts.

### Pre-conversion state

#### Laboratory equipment pre-conversion

Before the refurbishment, the lab was equipped with the UF1000 urinalysis system and VITEK 2 system (bioMérieux, France) for microbial identification.

#### Pre-conversion workflow

The urine sample workflow consisted of many manual steps, from triage to antibiotic sensitivity testing (AST) reporting. Urine samples were screened using the UF1000i (Sysmex, Japan) urinalysis system, which analyzed particles present in the urine by flow cytometry. If the result was suggestive of a positive urine sample, it was streaked onto conventional media and incubated for 24 h. If the result was suggestive of a negative urine sample, unless a culture was specifically requested by the physician, the sample was discarded without culture. After reading these plates, characteristic colonies were streaked onto CHROMID CPS Elite agar (bioMérieux, France) and incubated for 24 h. *Escherichia coli* was identified directly from CHROMID CPS Elite agar; other characteristic colonies were identified using VITEK2. AST was done using VITEK 2 unless other tests were requested.

Vaginal swab samples were received in Stuart medium and cultured on blood agar, Sabouraud, and selective agar containing vancomycin, colistin, amphotericin B, and trimethoprim plates. In case of suspicion of *Streptococcus agalactiae*, group B was requested, and the sample was streaked onto Strep B agar. Plates were incubated at 35°C–37°C for 48 h. Identification and AST were done on isolated colonies using VITEK 2 or manual methods.

Stool samples were received in Enteric Transport Medium and then cultured on Hektoen agar incubated for 48 h at 35°C–37°C, Campylosel agar was incubated for 48 h at 40°C–42°C, and selenite broth. Pathogens were subcultured onto conventional agar before identification and AST using VITEK 2.

### Actions taken post-automation

#### Lean assessment

bioMérieux Lab Consultancy experts applied lean methodology (14, 35) to assess the pre- and post-automation situation in the laboratory, including data collection from the laboratory information system, lab organization (sample volumes and types), staff scheduling, the sample arrival pattern, and positivity rates. Interviews with managers and technologists were performed and direct observation of selected points in the laboratory was carried out. The consultants used snapshots, spaghetti diagrams, volume vs staff analysis, process mapping, time-and-motion study, and brainstorming and problem-solving sessions. During the pre-conversion phase, this information was also used as input to develop the future lean layout of the laboratory capable of accounting for the laboratory’s future needs.

#### Change management sessions

To best manage the change in operating the JCS laboratory, the following actions were undertaken: assessment to measure the gap between the current practices and the expectations, customization to provide an adapted plan and tuned familiarization modules, and animation to deal with doubts and expectations of staff members and to support the transformation using five hands-on modules based on serious game methodology.

#### Continuous improvement events

bioMérieux Lab Consultancy experts ran two CIEs (also known as Kaizen events) after WASPLab and VITEK MS reached routine implementation in May and September 2021. CIEs focused on streamlining the workflows around the technology to reduce non-value-added tasks, remove bottlenecks, reduce waiting times, and standardize the non-automated steps. They were delivered following the steps of the A3 thinking lean problem-solving approach (understanding and alignment of the current problem and objectives to achieve, brainstorming and problem-solving to define root causes and potential countermeasures to be implemented, workshops and tests to implement and validate the change, data collection during Kaizen to validate current and future states). The tools applied comprised: 5S (sort, set in order, shine, standardize, sustain), one-piece flow, visual management, and standardized work. The changes implemented after CIEs led to streamlining the workflows based upon the time of sample arrival and standardized reading times per sample type by defining a standard work and displaying priorities on a per hour basis. It was reinforced by implementing a standard huddle 1 h before the end of either shift. This corresponded to two 5-minute team meetings to review current workload, adapting priorities if needed, and ensuring the standard work was followed. On top of these workflow optimization measures, CIEs led to standardization of bench organization and included the replenishment of benches in the standard work to avoid blockage during the activity peaks. After lab automation, samples were loaded continuously and read after incubation times defined by manufacturers’ recommendations. Characteristic colonies were identified using VITEK MS, and AST was done using VITEK 2 between 8 am and 10 pm. For urine samples, the standard work included a priority reading between 8 am and 11 am to launch a maximum number of ID and AST analyses before 11 am, in order to release a maximum number of results the same day as confirming positivity.

### Key performance indicators

Two main KPIs were analyzed: *productivity,* measured as the number of samples tested per FTE/day, and the *turnaround time,* defined as the time duration between sample receipt and the validation of all the microbiological documentation including AST and expressed as median and Q1–Q3 IQR. FTE corresponded to the employee’s scheduled hours divided by the employer’s hours for a full-time workweek. KPIs were measured for negative/positive urine, vaginal swab, and stool samples at three stages of the project, i.e., pre-conversion, post-automation, post-automation, and CIEs. JCS management defined KPIs to be reached following the automation and CIEs, after which, productivity of 100 samples/FTE/day was to be achieved. Desired TATs were defined at 20 h for negative and 40 h for positive urine samples, at 45 h for negative and 60 h for positive vaginal swab samples, and at 50 h for negative and 65 h for positive stool samples (Table 1).

### Statistical analysis

Because each period assessed was associated with large and multifactorial technological and organizational changes, median TATs were compared at consecutive timepoints using Mood’s median test, which does not assume any similarity or difference in group distribution shape. *P*-values of ≤0.05 were considered to indicate significant differences.
